# Perceived annoyance from environmental odors and association with atmospheric ammonia levels in non-urban residential communities: a cross-sectional study

**DOI:** 10.1186/1476-069X-11-27

**Published:** 2012-04-18

**Authors:** Victoria Blanes-Vidal, Esmaeil S Nadimi, Thomas Ellermann, Helle V Andersen, Per Løfstrøm

**Affiliations:** 1 Inst. Chemical Eng., Biotechnology and Environmental Tech., Faculty of Engineering, University of Southern Denmark, Odense, Denmark; 2 Dept. Environmental Science, Aarhus University, Roskilde, Denmark; 3 Dept. Construction and Health, Danish Building Research Institute, Aalborg University, Hørsholm, Denmark

**Keywords:** Odor, Waste, Slurry, Exposure, Livestock, Model

## Abstract

**Objective:**

Odor exposure is an environmental stressor that is responsible of many citizens complains about air pollution in non-urban areas. However, information about the exposure-response relation is scarce. One of the main challenges is to identify a measurable compound that can be related with odor annoyance responses. We investigated the association between regional and temporal variation of ammonia (NH_3_) concentrations in five Danish non-urban regions and environmental odor annoyance as perceived by the local residents.

**Methods:**

A cross-sectional study where NH_3_ concentration was obtained from the national air quality monitoring program and from emission-dispersion modelling, and odor pollution perception from questionnaires. The exposure-response model was a sigmoid model. Linear regression analyses were used to estimate the model constants after equation transformations. The model was validated using leave-one-out cross validation (LOOCV) statistical method.

**Results:**

About 45% of the respondents were annoyed by odor pollution at their residential areas. The perceived odor was characterized by all respondents as animal waste odor. The exposure-annoyance sigmoid model showed that the prevalence of odor annoyance was significantly associated with NH_3_ concentrations (measured and estimated) at the local air quality monitoring stations (p < 0.01,R^2^ = 0.99; and p < 0.05,R^2^ = 0.93; respectively). Prediction errors were below 5.1% and 20% respectively. The seasonal pattern of odor perception was associated with the seasonal variation in NH_3_ concentrations (p < 0.001, adjusted R^2^ = 0.68).

**Conclusion:**

The results suggest that atmospheric NH_3_ levels at local air quality stations could be used as indicators of prevalence of odor annoyance in non-urban residential communities.

## Background

Odor is an environmental pollutant that can impose physical, psychological, social and behavioral stress to humans. As a result, exposure to outdoor malodor in residential areas can cause negative public reactions and complaints from the citizens. Annoyance is the first negative reaction reported by humans exposed to increasing concentrations of environmental malodor, and it has been pointed out as an important component of an early warning system of health impairment [1]. Annoyance can be defined as “a feeling of displeasure associated with any agent or condition, known or believed by an individual or group to adversely affect them” [2]. People annoyed by odor may also report respiratory symptoms and health impairment even at odorant exposures below irritation thresholds as a result of psychological or stress mechanisms [3]. Odor annoyance means a significant degradation in the quality of life and the social well-being dimension of health, and it can be considered a problem even when only a small proportion of the population (5%) is bothered at rather infrequent occasions (2% of the time) [4].

Citizens expect rural air to have characteristic pleasant odors (e.g. freshly turned soil) or to be odorless [5]. However, during the last 50 years the livestock industry, typically located in rural areas, has followed an intensification process at both functional level (towards larger, more specialized and intensive livestock industrial systems) and spatial level (by geographic concentration of livestock production in specific areas with cheap input supplies and good market outlets) [6]. This intensification, together with population growth and residential development at the peri-urban and historically rural areas have increased the number of residents exposed to livestock odors [7]. Expectations of clean air and rural life style that are formed before settlement, and the trend for rural residents to have less tolerance to livestock odors and to be more demanding for quality of life, have made citizen complaints of odor annoyance from animal production to significantly increase during the past decades [8,9].

The US National Research Council [10] identified odor exposure in non-urban/agricultural areas as a major concern at the local level. However, odor pollution is difficult to assess and regulate, firstly, because olfactometric odor measurements are expensive and therefore, measurement campaigns are usually very limited in space and time. Secondly, because odor perception is a result of a complex mixture of odorant gases, which depends on the concentration of individual odorants and the existence of interaction effects between them [11]. Previous laboratory studies on odor perception in mixtures of odorants have revealed the existence of different types interaction effects: masking or dominant effect [12], averaging effect [13], hypoadditivity [14], normal additivity and hyperadditivity [15,16]. The use of analytical methods for odor assessment (in principle more reliable and cheaper than olfatometric methods), is limited by the fact that odor perceived by humans cannot be easily predicted from the concentration of individual compounds. Finally, because odor annoyance is a subjective and complex relation between a given gas concentration situation and a given individual, and this relation is affected by both sensory and non-sensory individual-specific factors of the exposed subject [17].

Many studies have attempted to identify a key odorous compound or compounds that can be related with odor from different odor sources [18-20]. Studies mainly differ on the type of experiment (i.e. laboratory, small scale or field experiments), the odor source (e.g. type and age of waste, management), the chemical analysis technique (e.g. gas chromatography–mass spectrometry, detection tubes, acid traps, photoaccoustic gas monitors, membrane inlet mass spectrometry, proton-transfer-reaction mass spectrometry), the odor characteristic assessed (e.g. odor concentration, odor intensity, odor index), and the sensory method (e.g. dilution to threshold olfactometry, gas chomatography-olfactometry). Regarding agricultural/livestock odors, some studies have found a relation between hydrogen sulfide (H_2_S) and odors, but others have identified other gases as main odorants, such as ammonia (NH_3_), volatile fatty acids, phenols or indoles [11,21-24]. In a recent investigation [24] including animal waste odorous air analysis by human panels and chemical analysis of NH_3_, H_2_S, and volatile organic compounds (VOCs), the authors concluded that NH_3_ was the only chemical odorant that significantly correlated with dynamic dilution olfactometry analysis with human panels, in the fresh (1 wk) and aged manure, and they identified NH_3_ as a key odorant as determined by chemical and gas cromatography-olfactometry. In another study [25], NH_3_, methyl mercaptan and dimethyl sulfide were identified as the key odor components significantly determining the odor index during slurry composting. McGuinn et al., [26] measured concentrations of dust and 14 odor-causing gases at increasing distances from four farms and showed that there was a positive relationship between NH_3_ concentration and odor intensity. Regarding studies with lower NH_3_ concentrations, Godbout et al. [27] showed that communities within swine production areas (151 animal units swine/km^2^) were exposed to higher NH_3_ and H_2_S concentrations, and also perceived higher odor intensities (assessed directly on site) compared to less producing areas.

National routine monitoring networks provide long-term, nationally consistent air quality data, that could potentially be used to assess and regulate rural air quality in relation to odor annoyance. However, information is needed to evaluate whether regional and temporal variations in gas measurements from air quality monitoring programs can be linked with regional and temporal variations in public perception of environmental odors. In this study we investigated the relationship (not causality) between ammonia (NH_3_) concentrations (measured by air quality monitoring stations and estimated from emission-dispersion models at the same locations) and odor annoyance perceived by non-urban local residents. Although odor is the result of a mixture of a large number of gases, NH_3_ concentration was chosen as a potential proxy of airborne exposure to gas and odors from livestock wastes, because:

(1) Approximately 80–90% of the total NH_3_ emissions in Western Europe and US originates from agricultural practices [28]. In Denmark, 97% of the NH_3_ emission is related to the agricultural sector and the main part, corresponding to 82%, is related to handling of manure whereas 3% relates to grazing animals [29]. Besides, the processes of slurry mixing, agitation and application which causes an acute increase of emissions of odors, also results in a sharp increase in NH_3_ emissions (among other gases) [30,31].

(2) Ammonia is also an odorous and irritating gas, which has certain contribution to livestock odor and has shown correlation with livestock odor as assessed by trained panelists in previous studies [11,24-26].

(3) The development of an NH_3_ exposure-odor annoyance model is of particular interest, because NH_3_ is usually part of long-term, routine monitoring networks designed to determine effectiveness of national air pollution programs and to provide information on status and trends in regional air quality, and so, extensive data on atmospheric NH_3_ concentrations is available in many European countries and in US.

The objectives of this study were: (1) To evaluate the association between NH_3_ concentrations measured and estimated at non-urban air quality monitoring stations, and prevalence of odor annoyance in local residential communities, and (2) To study the link between seasonal patterns in NH_3_ measurements and public perception of the seasonal variation on odor pollution.

## Methods

### Ammonia concentration

**Figure 1:**
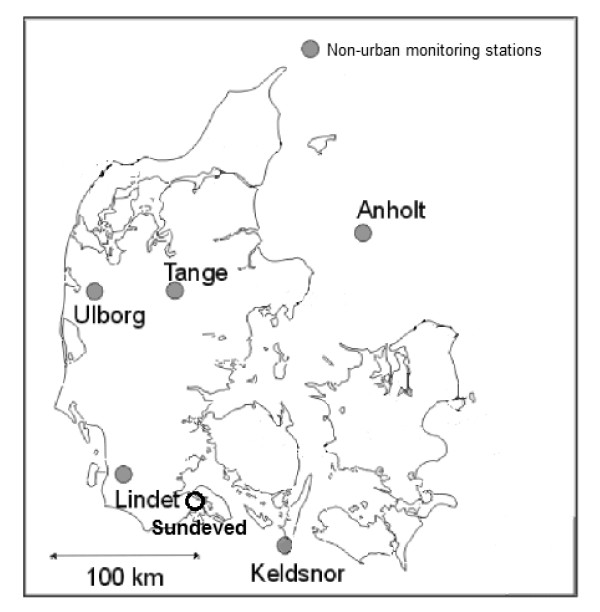
Danish air quality monitoring stations: Anholt (Region I), Ulfborg (Region II), Keldsnor (Region III), Tange (Region IV) and Lindet (Region V). Location of Sundeved (Region VI).

The Danish National Air Quality Monitoring Programme includes a nation-wide network of air pollution monitoring stations that gives a national geographical coverage of a basic set of air pollutants. Five of these stations are located in non-urban areas and are equipped with semi-automatic filter pack samplers to measure NH_3_ concentrations in the air on a daily basis. These five stations are: Anholt (Region I), Ulfborg (Region II), Keldsnor (Region III), Tange (Region IV) and Lindet (Region V) (Figure 1). The specific filter pack method used in this study consists of a series of three filters: a particle filter (mixed esters of cellulose MF-Millipore RA, l 2/an, 50 mm) followed by two filters Whatman 41 (50 mm), one of them impregnated with oxalic acid for NH_3_ collection. The filter holder was made of polycarbonate with PVC inlet. The flow was about 40 l/min (at 0°C), kept constant by electronic regulation. The inlet was placed 2 m above ground. Heating of the filters by sunshine was minimized by placing the filter holder in a reflecting metal shield with about 5 cm of the PVC inlet unshielded. Following the exposure, all filters were extracted in 20 ml deionized water. Ammonia absorbed on the oxalic acid filter was analyzed as NH_4_^+^ according to the continuous flow analysis method described in DS/EN ISO 11732. The precision of the filter pack measurements is 10-20%. The detection limit for a 24 h exposure, defined as three times the standard deviation of the blanks, is 0.04 μg NH_3_-Nm^-3^.

The five regions where the monitoring stations are located represent areas with different degree of agricultural activity and therefore NH_3_ emission rates. Animal densities at the municipalities of regions I, II, III, IV and V are: 0, 5.2, 3.8, 7.9 and 2.7 swine/ha, respectively, and 0, 0.9, 0.6, 0.7, 1.1 cattle/ha. The proportion of agricultural land is 0, 60%, 73%, 60% and 82%, respectively [32]. The station Anholt (Region I) is located in a small island (20 km^2^) situated more than 50 km from the continent and with no local sources of NH_3_. The station Ulfborg (Region II) is surrounded by forest, heath land and small agricultural areas. The station Kelsnor (Region III) is placed at the east coast line of the island Langeland. Agricultural activity is found close to the station only in the western direction. The station Tange (Region IV) is surrounded by agricultural land, grassland, wetland and a lake. The station Lindet (Region V) is located in a forest clearing and the forest is surrounded by farmland and heavy agricultural activity. All the NH_3_ monitoring sites are located more than 500 m away from high NH_3_ emission sources, so that the effect of local/hotspot emissions is avoided or minimized.

Emissions-based atmospheric dispersion modeling allows estimating NH_3_ concentrations in a region regardless of the existence of an air quality station. Ammonia concentrations in 12km × 12 km areas surrounding these five monitoring stations (regions I to V) and in an additional sixth area (region VI), were estimated by emission/dispersion modeling, with a spatial resolution of 400mx400 m. The sixth area (Region VI, Sundeved) was included in the study to get more insight about odor annoyance in a highly intensified agricultural area. Swine density in Region VI is the highest in the country (i.e. 13.1 swine/ha) and 82% of its surface is agricultural land. Ammonia concentrations in the six areas were estimated by combining information from two models: The Danish Eulerian long-range transport model (DEHM) and the local-scale transport deposition model (OML-DEP) [33]. Both models use meteorological data generated by the Fifth-Generation Penn State/NCAR Mesoscale Model. The DEHM model was used to estimate the background NH_3_ concentration at each region. The OML-DEP model was used to calculate NH_3_ dispersion from local point sources and surface sources. The OML-DEP model is a Gaussian dispersion model based on the boundary-layer theory, which also accounts for chemical transformation of NH_3_ to NH_4_^+^. In the OML-DEP a detailed emission inventory was used for fields (area sources), and stables and animal waste storages (which were considered as point sources). Concentrations were calculated in a regular grid of 400 m × 400 m. Ammonia concentrations at locations between grid-points were estimated from multivariate interpolation by inverse distance weighting (IDW) from modeling results.

### Cross-sectional questionnaire data

Information on perceived air quality was collected by means of questionnaires. A total number of 470 households within the six study regions were randomly selected. Twenty of the households were located in Region I, while 450 households were located in regions II, III, IV, V and VI (90 households/region). Questionnaires were mailed when field application of animal slurry is restricted by law in Denmark (from November to February months). Adults (>18 years old) living at the household were requested to fill and return the questionnaire, which was presented as a general survey on living conditions in the countryside. Participants gave their informed consent. The structured questionnaire started with an open-ended question whereby participants listed according to their own experience the main advantages and disadvantages of living in the countryside. Questions regarding odor pollution included: degree of perceived annoyance (i.e. Not annoying = 0, Slightly annoying = 1, Moderately annoying = 2, Very annoying = 3, and Extremely annoying = 4), season of highest perceived annoyance (i.e. winter, spring, summer and autumn) and origin of odor (i.e. traffic, industry, farm, livestock waste spreading, unknown, or others). Additional socio-demographic data were included.

### Statistical analysis

An exposure-response model was developed for the relation between atmospheric NH_3_ concentrations and odor annoyance responses at the study regions. The exposure-response model was a sigmoid model (Equation 1), based on Nicell et al. [34], who showed that individual thresholds for odor annoyance in a population are log-normally distributed:

(1) $$A=\frac{1}{1+e^{a\cdot \log_{e}C+b}}$$

Where A is the proportion of the population annoyed by odor, C is the NH_3_ concentration (μg/m^3^) and a and b are constants. Linear regression analyses were used to estimate the model constants after equation transformations. The exposure-response model was validated using leave-one-out cross validation (LOOCV) statistical method which compared prevalence obtained from questionnaire data and values predicted by the LOOCV of the exposure-response models.

Differences among regions regarding NH_3_ concentrations and socio-demographic characteristics of the respondents were evaluated by Generalized linear model (GLM) statistical analyses. All statistical analyses were performed in R.

## Results

### Participants demographics and ammonia concentrations

About 38% of the approached households (180 subjects) agreed to participate in the study. Table 1 shows the socio-demographic characteristics of the participants stratified by region and in total. The statistical analyses showed no significant differences among individuals from the different regions (Table 1).

**Table 1:** Frequency distribution and socio-demographic characteristics of the participants stratified by region and in total

	**Region**						
	**I**	**II**	**III**	**IV**	**V**	**VI**	**Total**
Gender^[a]^							
*Male*	64 (7)	53 (17)	67 (20)	34 (11)	48 (15)	50 (22)	51 (92)
*Female*	36 (4)	47 (15)	33 (10)	66 (21)	52 (16)	50 (22)	49 (88)
Age (years)^[b]^	57 ± 8	53 ± 15	59 ± 12	47 ± 14	52 ± 15	54 ± 16	54 ± 16
Current smoking habit^[a]^							
*Yes*	27 (3)	16 (5)	17 (5)	16 (5)	6 (2)	61 (27)	26 (47)
*No*	73 (8)	84 (27)	83 (25)	84 (27)	94 (29)	39 (17)	74 (133)
Childhood living environment^[a]^							
*Countryside*	18 (2)	47 (2)	37 (15)	38 (11)	48 (12)	39 (15)	40 (72)
*Village*	36 (4)	34 (11)	23 (7)	41 (13)	26 (8)	30 (13)	31 (56)
*Town or city*	45 (5)	19 (6)	40 (12)	22 (7)	26 (8)	32 (14)	29 (52)
Years living in the area^[b]^	19 ± 14	31 ± 19	31 ± 20	26 ± 19	27 ± 19	31 ± 20	29 ± 19
Children living in the household^[a]^							
*No children*	91 (10)	69 (22)	80 (24)	56 (18)	55 (17)	61 (27)	66 (118)
*A least one child <2 years old*	9 (1)	3 (1)	3 (1)	16 (5)	19 (6)	5 (2)	9 (16)
*A least one child 2–10 years old*	0 (0)	13 (4)	7 (2)	22 (7)	6 (2)	18 (8)	13 (23)
*A least one child 11–18 years old*	0 (0)	16 (5)	10 (3)	6 (2)	19 (6)	16 (7)	13 (23)
Time spent at home (h/wk)^[b]^	129 ± 30	114 ± 31	124 ± 38	114 ± 33	110 ± 33	109 ± 48	115 ± 38
Current job^[a]^							
*Agriculture related*	0 (0)	6 (2)	3 (1)	3 (1)	6 (2)	7 (3)	5 (9)
*Not-agriculture related*	100 (11)	94 (30)	97 (29)	97 (31)	94 (29)	93 (41)	95 (171)

^[a]^% (number of respondents).

^[b]^ mean ± standard deviation.

Measured and modeled NH_3_ concentrations increased with the level of agricultural activity in the region, from Region I to Region V (Table 2). Annual averaged NH_3_ concentrations measured at the air quality monitoring stations ranged from 0.16 μg/m^3^ (Region I) to 1.34 μg/m^3^ (Region V), while modeled NH_3_ concentrations at the same locations ranged from 0.15 μg/m^3^ (Region I) to 1.54 μg/m^3^ (Region V). Emissions-based atmospheric dispersion modeling data showed that residential NH_3_ exposure is highly variable within the areas. However, in general terms, respondents living in regions with more animal production and agricultural activities (i.e. regions IV, V and VI) were exposed to higher concentrations of NH_3_ at their residences than respondents living in less intensive agricultural areas (Table 2). More information regarding the local-scale association between household-specific outdoor concentrations, person-related variables and annoyance responses can be found in [17].

**Table 2:** **Ammonia (NH**_**3**_**) concentrations measured and modelled at air quality stations and summary of modelled NH**_**3**_**concentrations at the residences**

**Region**	**NH**_**3**_**concentration at the air quality station (μg/m**^**3**^**)**		**Number of respondents (and %) exposed to different modelled NH_3_ concentration categories at their residences (μg/m**^**3**^**)**							
	**Measured**^**[a]**^	**Modeled**^**[b]**^	**0-0.5**	**0.5-1**	**1-1.5**	**1.5-2**	**2-2.5**	**2.5-3**	**3-3.5**	**>3.5**
I	0.16 ± 0.00^a^	0.15 ± 0.01^a^	11 (100)	0	0	0	0	0	0	0
II	0.62 ± 0.03^b^	0.85 ± 0.15^bc^	0	7 (22)	14 (44)	8 (25)	3 (9)	0	0	0
III	0.53 ± 0.05^b^	0.90 ± 0.12^c^	0	13 (43)	10 (33)	4 (13)	1 (3)	1 (3)	0	1 (3)
IV	1.02 ± 0.02^c^	1.27 ± 0.16^cd^	0	0	8 (25)	8 (25)	10 (31)	3 (9)	3 (9)	0
V	1.34 ± 0.10^d^	1.54 ± 0.01^d^	0	0	0	1 (3)	18 (58)	6 (19)	1 (3)	5 (16)
VI	N/A	N/A	0	0	0	0	12 (27)	9 (20)	14 (32)	9 (20)

^[a]^ Annual averaged NH_3_ concentration measured at air quality stations in 2008 and 2009, average ± standard deviation.

^[b]^ NH_3_ concentration from emission-dispersion models at the location of the air quality station in 2008 and 2009, average ± standard deviation.

Same letters within columns indicate no significant differences (*P* > 0.05).

### Prevalence of odor annoyance

About 46% of the respondents (83 subjects) were annoyed by odor pollution at their residences, being 60 subjects “slightly annoyed”, 11 “moderately annoyed”, 8 “very annoyed” and 4 “extremely annoyed” (Table 3). The perceived odor was characterized by all respondents as animal waste odor. Higher odor annoyance prevalence was reported in regions with higher degree of agricultural intensification (i.e. Regions IV, V and VI).

**Table 3:** Prevalence of odor annoyance expressed as the percentage (%) and number (N) of respondents at each region reporting odor annoyance

**Region**	**Not annoyed** **(score = 0)**		**Slightly annoyed** **(score = 1)**		**Moderately annoyed** **(score = 2)**		**Very annoyed** **(score = 3)**		**Extremely annoyed** **(score = 4)**	
	%	N	%	N	%	N	%	N	%	N
I	100	11	0	0	0	0	0	0	0	0
II	66	21	34	11	0	0	0	0	0	0
III	73	22	20	6	0	0	7	2	0	0
IV	47	15	28	9	19	6	6	2	0	0
V	32	10	52	16	10	3	6	2	0	0
VI	41	18	41	18	5	2	5	2	9	4

**Figure 2:**
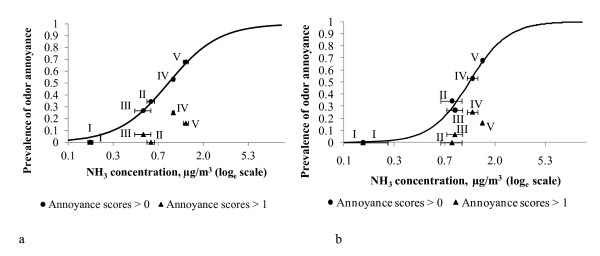
**Relationship between prevalence of odor annoyance at community level and NH**_**3**_**concentrations at air quality stations: measured (Figure**2**a)and from emission-dispersion modelling (Figure**2**b).** Prevalence of odor annoyance is expressed as the proportion of respondents at each region that reports annoyance scores > 0 (odor annoyance at any degree) and annoyance scores > 1 (moderately, very or extremely annoyed by odor). Error bars indicate coefficients of variation of NH_3_ concentrations.

The exposure-response model at regional level (Equation 1) showed that averaged NH_3_ concentration measured in the local air quality monitoring stations was significantly associated with the prevalence of odor annoyance in the region at any degree (residents being slightly, moderately, very or extremely annoyed by odor, i.e. annoyance scores > 0) (Figure 2). The fitted constants and associated standard errors in Equation 1 were: a_s_ = −1.806 ± 0.092; b_s_ = −0.165 ± 0.039 (p < 0.01; adjusted R^2^ = 0.99). Similar results were obtained for the relationship between NH_3_ concentration at the air quality station estimated from dispersion modeling and odor annoyance responses (annoyance scores > 0) (a_m_ = −2.642 ± 0.523; b_m_ = 0.463 ± 0.142 (p < 0.05; adjusted R^2^ = 0.93). The positive association between ammonia concentrations and the percentage of respondents showing higher degrees of annoyance (moderately, very or extremely annoyed by livestock odors, i.e. annoyance scores > 1) that is observed in Figure 2, was not statistically significant (p > 0.05). The leave-one-out cross validation (LOOCV) showed that the exposure-response models predicted annoyance prevalence at each region with a maximum relative error of 5.1% (from measured NH_3_) and 20% (from modelled NH_3_) (Table 4).

**Table 4:** Leave-one-out cross validation (LOOCV) of the exposure-response model

**Validation region**	**Measured annoyance prevalence**^**[a]**^	**LOOCV from NH**_**3**_**measurements**^**[b]**^			**LOOCV from NH**_**3**_**estimations**^**[c]**^		
		**Predicted annoyance prevalence**	**Absolute error**	**Relative error,%**	**Predicted annoyance prevalence**	**Absolute error**	**Relative error,%**
I	0	0.04	0.04	-	0.01	0.01	-
II	0.34	0.33	−0.02	−5.1	0.27	−0.07	−20
III	0.27	0.28	0.01	3.3	0.25	−0.02	−6.9
IV	0.53	0.56	0.03	5.1	0.42	−0.11	−20
V	0.68	0.65	−0.03	−4.5	0.56	−0.12	−18

^[a]^ Expressed as the proportion of respondents annoyed by odors at each validation region, as obtained from questionnaire responses.

^[b]^ R^2^ > 0.99 and p-value < 0.05 in all models.

^[c]^ R^2^ > 0.93 and p-value < 0.05 in all models.

In general terms, the prevalence of odor annoyance was in agreement with the responses to the open-ended question regarding the main advantages and disadvantages of living in the countryside. About 22% of the respondents mentioned “clean air” as one of the main advantages of rural life. This percentage was lower in regions with more intensive farming and agricultural activities (i.e. 36%, 31%, 20%, 22%, 23% and 16% of respondents from regions I, II, III, IV, V and VI, respectively). About 6% of the respondents spontaneously reported “animal waste odor” as one of the main disadvantages, being all of them from regions III (3%), IV (13%), V (6%) and VI (7%).

### Seasonal variation in atmospheric ammonia levels and perceived odor annoyance among residents

**Figure 3:**
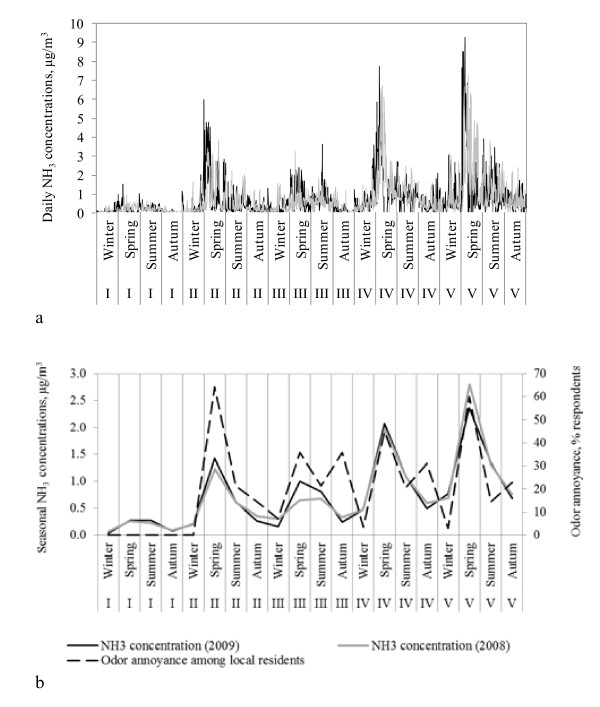
**Measured NH**_**3**_**concentrations and odor annoyance among non-urban residents in regions I to V.** Figure 3a. Daily measured concentrations. Figure 3b. Averaged seasonal concentrations and odor annoyance. The opposite trends in odor annoyance and NH_3_ emissions observed in autumn in regions III, IV and V are explained by the fact that application of slurry in autumn in Denmark is not generalized as it occurs in spring. Responses on odor annoyance in autumn are more sensitive to the existence (or absence) of winter crops in the proximity of the specific respondents’ houses. Excluding these three data points from the regression analysis between seasonal variations of NH_3_ concentrations and perceived odor annoyance at each region, increases the coefficient of determination from R^2^ = 0.68 to R^2^ = 0.91.

Daily and seasonal NH_3_ concentrations measured at the five air quality stations are shown in Figure 3. Ammonia concentrations during spring and summer seasons were 85 ± 25% and 20 ± 26% higher than annual averages at each region (mean ± standard deviation), while NH_3_ concentrations during autumn and winter were lower than annual averages (−47 ± 2% and −58 ± 10%, respectively).

Odor annoyance was mostly experienced during spring season (51% of the respondents), followed by autumn (26%), summer (19%) and winter (4%). About 27% of the residents annoyed by livestock odors reported to be annoyed only in spring season, while 18% reported to experience odor annoyance in both spring and autumn seasons. Each of the remaining possible combinations were reported by less than 6% of the respondents. Only 2% of the respondents annoyed by livestock odors experienced odor annoyance during all four seasons. Regression analysis between seasonal variations of NH_3_ concentrations and perceived odor annoyance at each region were statistically significant (p < 0.001, adjusted R^2^ = 0.68).

**Figure 4:**
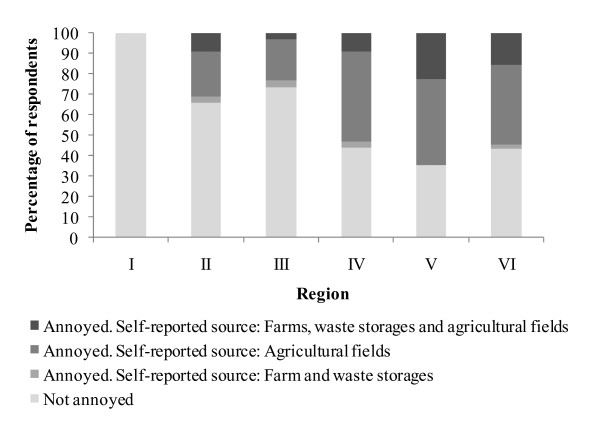
Self-reported sources of odors causing annoyance as identified by non-urban residents.

Responses from the local residents regarding the source of odors causing annoyance showed that the majority of respondents (i.e. 70% of the respondents that reported to experience odor annoyance) identified agricultural fields (where animal wastes are applied as fertilizer) as the only source of odor at their residences (Figure 4). Only 5% of the respondents annoyed by odors reported that farms and waste storage units, were the only sources of odor at their residences. Finally, about 25% of the respondents annoyed by odors reported that the origin of odors at their residences were the emissions from farms, waste storage units and agricultural fields.

## Discussion

More than 168 volatile odorous compounds have been identified in animal wastes and in the air around them [35]. In order to evaluate and reduce odor nuisances in non-urban residential areas, it is important to determine what compounds can be used as odor markers and to develop exposure-response models. Our results showed that atmospheric NH_3_ concentration was associated with odor annoyance perceived by non-urban residential communities. This association occurred both when NH_3_ concentration was obtained from standard national air quality monitoring stations and when NH_3_ concentration at the same location was estimated from emission-dispersion models. Odor is the result of a complex mixture of many gases, and livestock odor can be detected even when the vast majority of chemical compounds are present at concentrations below published odor detection thresholds, due to the existence of cumulative effects [36]. Although some laboratory experiments have shown that the contribution of NH_3_ to the perceived animal waste odor can be limited, other studies have found that NH_3_ concentrations can show significant correlations with animal waste odor as assessed by trained panelists [11,24-26]. The NH_3_-odor annoyance association can be also explained by the fact that animal wastes are the main source of both odor and NH_3_ emissions in non-urban regions [28,29] and that the process of slurry mixing, agitation and application (which is the origin of most odor complaints from the local citizens [37,38]) also causes an acute increase of NH_3_ emissions [30,31]. The result of our study is especially significant because it may allow developing and establishing cost-effective strategies for odor assessment and regulation based on NH_3_ concentration measurements. However, future studies in more locations and scenarios are required to assess the general validity of the results.

Most odorous compounds produced and emitted from animal wastes are the result of an incomplete anaerobic decomposition which may occur during waste collection, handling, storage, and land application, but the contribution of each activity to the total emission of odor and odorants remains unclear. Previous studies have suggested that fields where animal waste is applied as fertilizer are responsible of the majority of odor complains in rural areas [37,38]. Wastes application to agricultural fields in Denmark may only take place during the growing season (i.e. 1st February to harvest for most crops and from harvest to 1^st^ October for winter crops), as the rest of the year slurry application is banned by Danish policies aimed at reducing nitrogen emissions from agriculture (Action Plan for Sustainable Agriculture). The seasonal patterns in measured NH_3_ concentrations are the result of these environmental policies, which causes that most of the slurry application in Demark (i.e. 93% of swine wastes and 79% of cattle slurry, [39]) and the NH_3_ emissions [40] occur in spring season. In our study, the subjective perception of local residents regarding the source of livestock odors and the fact that odor annoyance perceived by local residents over the course of the year is related to the seasonal patterns of field application of animal wastes, suggests that agricultural lands are the main source of odor in rural areas. Odor annoyance during spring and summer may also be enhanced by the effect that outdoor odor may have on the behaviour of the local residents, preventing them from performing outdoor activities during the warmer seasons of the year [17,41].

## Conclusions

One of the main challenges for the development and application of odor policies in non-urban residential areas is the identification of a single easy-measurable gas that can be used as a marker of a complex mixture of odorant and irritant chemicals that causes annoyance and affects the well-being and health of non-urban residents. This study provides evidences that suggest that NH_3_ concentration measured and modeled as part of the national air quality programs could be used as proxy of prevalence of odor annoyance in non-urban residential communities. Regional and seasonal variations in measured NH_3_ concentrations were associated with odor annoyance experienced by non-urban citizens. The results are especially significant because NH_3_ is usually part of long-term, routine monitoring networks in many European countries and in the US, which could potentially be used to assess and regulate rural air quality in relation to odor annoyance. Future studies are required to assess the general validity of the results.

## Competing interests

The authors declare that they have no competing interest.

## Authors’ contributions

VBV conceived and coordinated the study, designed the study, collected data, performed data analysis and drafted the manuscript; ESN participated in the data collection and analysis and provided reviews of the text, TE, HVA and PL provided air pollution information and provided reviews of the text. All authors have read and approved the final manuscript.
